# Hepatic Steatosis, Rather Than Underlying Obesity, Increases the Risk of Infection and Hospitalization for COVID-19

**DOI:** 10.3389/fmed.2021.636637

**Published:** 2021-03-29

**Authors:** Adriana Roca-Fernández, Andrea Dennis, Rowan Nicholls, John McGonigle, Matthew Kelly, Rajarshi Banerjee, Amitava Banerjee, Arun J. Sanyal

**Affiliations:** ^1^Perspectum Ltd., Oxford, United Kingdom; ^2^University College London Hospitals National Health Service Trust, London, United Kingdom; ^3^Institute of Health Informatics, University College London, London, United Kingdom; ^4^Barts Health National Health Service Trust, The Royal London Hospital, London, United Kingdom; ^5^Division of Gastroenterology, Hepatology, and Nutrition, Virginia Commonwealth University School of Medicine, Richmond, VA, United States

**Keywords:** liver fat, COVID-19, risk factor, biomarker, disease severity

## Abstract

**Objective:** Obesity is a risk factor for SARS-COV2 infection and is often associated with hepatic steatosis. The aim of this study was to determine if pre-existing hepatic steatosis affects the risk of infection and severity for COVID-19.

**Design:** Prospective cohort study (UK Biobank). Univariate and stepwise multivariate logistic regression analyses were performed on liver phenotypic biomarkers to determine if these variables increased risk of testing positive and being hospitalized for COVID-19; then compared to previously described risk factors associated with COVID-19, including age, ethnicity, gender, obesity, socio-economic status.

**Setting:** UK biobank study.

**Participants:** 502,506 participants (healthy at baseline) in the UK Biobank, of whom 41,791 underwent MRI (aged 50–83) for assessment of liver fat, liver fibro-inflammatory disease, and liver iron. Positive COVID-19 test was determined from UK testing data, starting in March 2020 and censored in January 2021.

**Primary and Secondary Outcome Measures:** Liver fat measured as proton density fat fraction (PDFF%) MRI and body mass index (BMI, Kg/m^2^) to assess prior to February 2020 using MRI of the liver to assess hepatic steatosis.

**Results:** Within the imaged cohort (*n* = 41, 791), 4,458 had been tested and 1,043 (2.49% of the imaged population) tested positive for COVID-19. Individuals with fatty liver (≥10%) were at increased risk of testing positive (OR: 1.35, *p* = 0.007) and those participants with obesity and fatty liver, were at increased risk of hospitalization with a positive test result by 5.14 times (*p* = 0.0006).

**Conclusions:** UK Biobank data revealed obese individuals with fatty liver disease were at increased risk of infection and hospitalization for COVID-19. Public policy measures and personalized medicine should be considered in order to protect these high-risk individuals.

## Strengths and Limitations of This Study

- The use of MRI liver biomarkers for measuring fat (Proton Density Fat Fraction, PDFF) and fibroinflammation (cT1 mapping) which have been proved to be excellent tools for characterizing liver disease and predicting clinical outcomes.
- The availability of PDFF and cT1 markers in the UKBB allows the characterization of liver tissue at population level that would not be possible with biopsy.
- Despite the relatively small number of positive test results, and the relatively small subset of patients with liver imaging, the UK Biobank is a large, long-term biobank study that provides with sufficient control data and MRI data to characterize liver disease before COVID-19.
- Restrictions in terms of clinical history available (blood biomarkers at the time of the MRI, information on symptoms during the acute phase of COVID-19 infection etc.,).

## Introduction

Coronavirus disease (COVID-19) is caused by the SARS-CoV-2 virus. Risk factors leading to severe infection have been identified in previous studies around the world (1–3) including increased age, male sex, non-Caucasian ethnicity, and the presence of pre-existing conditions, in particular, cardiovascular and metabolic conditions, which affect liver function (4, 5).

The majority of patients experiencing COVID-19 develop abnormal liver function (6–8), with studies reporting abnormal alanine transaminase (ALT), aspartate aminotransferase (AST), albumin levels, and increased gamma-glutamyl transferase (GGT) in severe patients (7). The development of liver injury has been more common in severe COVID-19, with liver injury in mild disease often transient and resolving without treatment.

The mechanistic link between COVID-19 and the liver may relate to the expression of the ACE2 protein, through which SARS-CoV-2 is believed to enter cells (9) in cholangiocytes (10). However, recent studies have also shown a hepatocellular profile of damage rather than cholestasis (11). Alternative hypotheses suggest that liver injury in severe cases is being caused by an immune response triggered by the infection (7) or due to a drug-induced liver injury from the antivirals, antibiotics and steroids used for treating COVID-19 (12).

Traditionally, characterization of chronic liver disease has relied on invasive liver biopsy to evaluate the pathological hallmarks of disease: fibrosis, inflammation, and steatosis (13). More recently, scalable, non-invasive alternatives using MRI have emerged as promising biomarkers for measuring liver fat (Proton Density Fat Fraction, PDFF), and liver fibroinflammation (cT1 mapping) which have demonstrated value in characterizing liver disease (14, 15) and predicting clinical outcomes (16). This has enabled determination of the current prevalence of fatty liver disease in the UK adult population, along with genetic associations with liver fat and fibroinflammation (17). Non-alcoholic fatty liver disease (NAFLD) is the term for a range of conditions caused by an excess of fat in the liver. With a high prevalence in overweight or obese people, NAFLD is also associated with an increased risk of other comorbidities as diabetes, high blood pressure or kidney disease. When the excess of fat in the liver is higher than 10%, NAFLD progresses into causing inflammation and scarring. Chronic steatosis may lead to liver fibrosis and to even more serious complication as such as liver cirrhosis.

The UK Biobank is a large-scale biomedical database in the United Kingdom investigating the development of disease, exploring both genetic predisposition, and environmental exposure, with COVID-19 testing data available.

We tested the hypothesis that comorbid liver disease, specifically liver fat accumulation, is an additional and independent risk factor infection and hospitalization for COVID-19.

## Methods

A retrospective analysis of the UK Biobank (UKB) was performed. The UKB is a population-based study with 502,506 individuals who have been deeply phenotyped and genotyped and are being followed prospectively to inform development of Precision Medicine in general. The UKB has approval from the North West Multi-Center Research Ethics Committee (MREC) and obtained written informed consent from all participants prior to the study. The data were extracted from the UKB under the application 9,914, in an anonymized dataset and did not meet criteria for being considered research requiring independent IRB approval and consent from the participants. The authors had access to all of the data and take responsibility for the contents of this manuscript. Due to the nature of this data there was no Patient and Public Involvement in this study.

### Study Population

The inclusion criteria included those who had a liver MRI prior to February 2020 using access application 9,914. There were no exclusion criteria.

### Data Acquisition

Patient meta-data including information on COVID-19 testing status, demographics, diabetes status, cardiometabolic risk factors, socio economic status, and genetic variants associated with liver disease were available, however, concomitant biochemistry data were not. COVID testing data was available from March 2020 to January 2021, results were censored in January 2021.

A liver fat (PDFF) over 5% was considered to represent hepatic steatosis (18). Severe steatosis was categorized by liver fat above 10%, a common threshold used in clinical trials of non-alcoholic steatohepatitis (NASH) (19). A liver cT1 >825 ms has been shown to predict liver-related clinical outcomes (16), comparably to histological fibrosis staging.

### Analytic Approach

The goal of this study was to test the hypothesis that pre-existing hepatic steatosis is a risk for having a positive COVID-19 test. Previously reported risk factors for COVID-19 which included being male, non-Caucasian ethnicity, being obese and having comorbid hypertension (20) as well as measures of socio-economic status (level of education and Townsend deprivation index) were also explored. Given the nature of how UKB data is acquired, the anthropometric features in this study were recorded at the first visit, at which a full set of data was available, and not at the time of the MRI scanning.

Based on the previous results, we also explored whether liver fat could influence the effect on BMI on hospitalization risk using stepwise multivariate analysis to explore exposures related to being hospitalized. This analysis showed a significant association for the interaction between BMI and high liver fat (≥10% PDFF) so to explore this relationship further, we created a new variable called “hepato-metabolic risk,” in which participants were stratified into four groups based on obesity and liver fat status and ran a multivariate logistic regression (Figure 4): group A (liver fat <10%, BMI <30 kg/m^2^), group B (liver fat ≥10%, BMI <30 kg/m^2^), group C (liver fat <10%, BMI ≥ 30 kg/m^2^), and group D (liver fat ≥10%, BMI ≥ 30 kg/m^2^).

### Statistical Analysis

Statistical analysis was performed using R software (version 3.6.1) with a *p*-value < 0.05 considered statistically significant. Descriptive statistics were used to summarize baseline participant characteristics. Mean and standard deviation (SD) were used to describe normally distributed-continuous variables, median with interquartile range for non-normally distributed, and frequency and percentage for categorical variables. Mean difference in biomarker values between those who tested positive for COVID-19 and those who did not were compared using the Wilcoxon test, and difference in the counts of the binary outcomes were compared using two tailed Fisher's exact test.

Analysis of the associations between all exposures and testing positive for COVID-19 was performed using univariate and stepwise multivariate logistic regression models. Additional multivariate logistic regression were also performed for (i) all the known risk factors and liver fat with an interaction term combining BMI and liver fat based on a priori assumption of a co-linearity between the exposures and (ii) all the known risk factors without liver fat to assess the significance of liver fat in the model. ANOVA was performed to determine if one model was superior to another. Both univariate and stepwise multivariate logistic regression were performed using the GLM function in R and the *brglm2* tool that aims to reduce the bias observed when results are sparse. Risk scores were extracted from the odds ratio estimates of having a positive rest as calculated in the logistic regression model with confidence intervals calculated with the Wald's test. In a second part of the analysis, we repeated the same procedure to define risk of hospitalization with a positive COVID-19 test. Hospitalization or admission to intensive care unit due to COVID-19 disease was defined as admission date 1 week before or 1 month after a positive test result.

## Results

**Figure 1:**
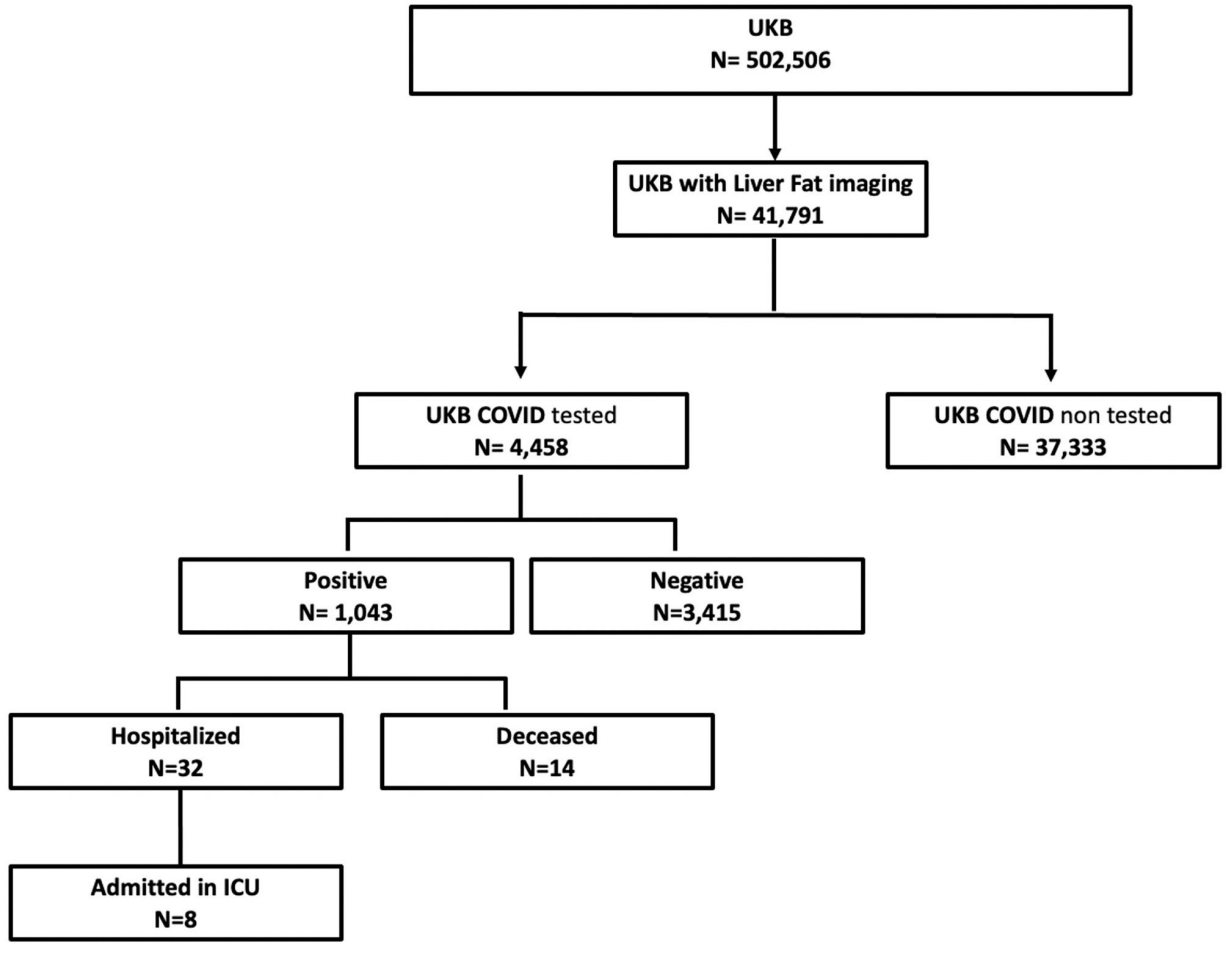
Summary of UKB data available.

**Figure 2:**
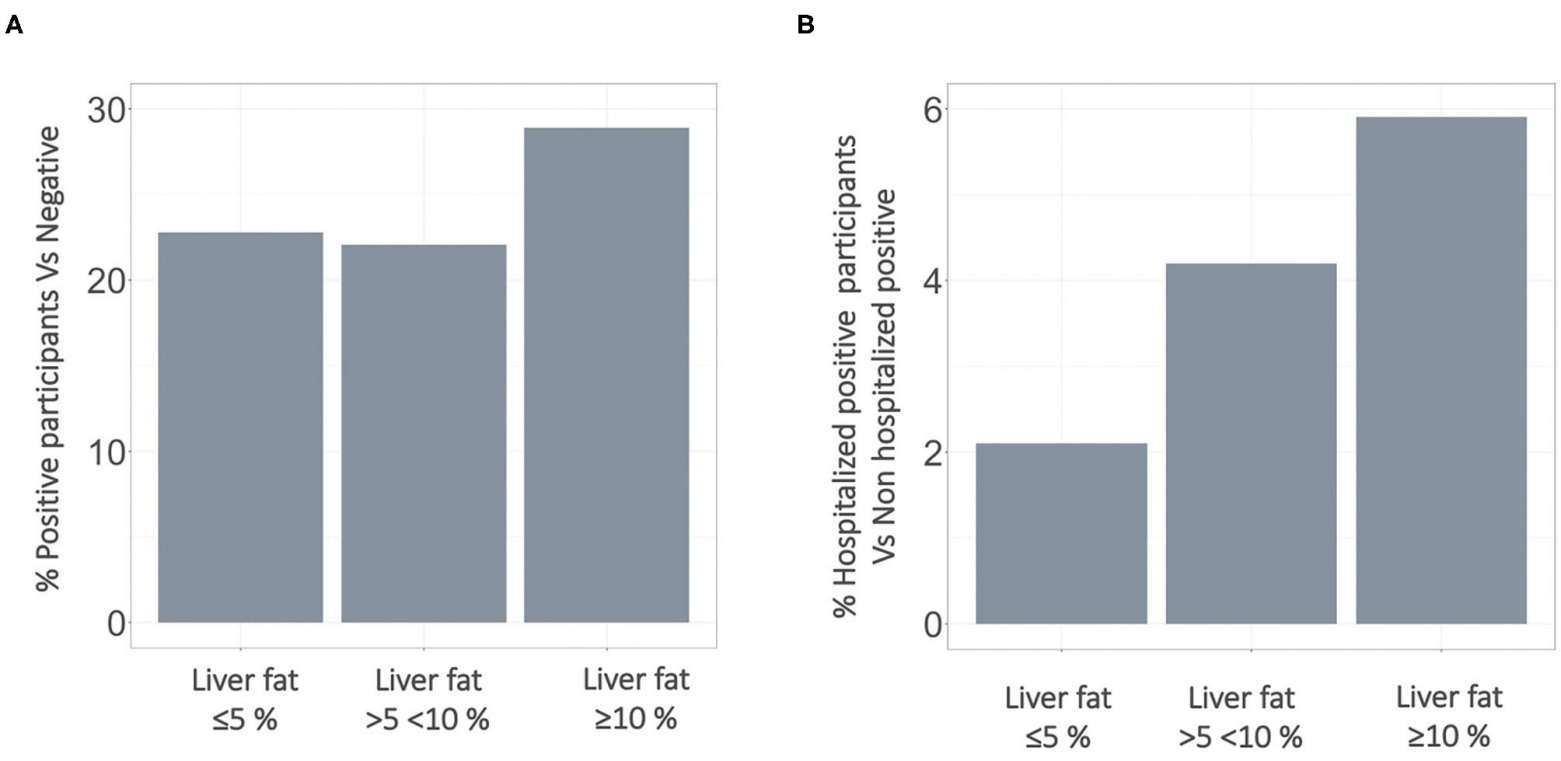
Stepwise increase in percentage of **(A)** participants testing positive for COVID-19 and **(B)** being hospitalized with a positive test result. Stratification based on pre-existing fatty liver disease. COVID-19, Coronavirus disease.

41,791 participants were extracted from the UK Biobank (UKB) imaging study. Of these, 4,458 had a COVID-19 test result available with 1,043 testing positive and 3,415 testing negative (Figure 1). Participant characteristics for each of the groups evaluated in this study are reported in Table 1. Comparing those that tested positive to the population testing negative, there was a higher percentage of non-Caucasian participants in the COVID-19 positive group, moreover, the positive group showed a significantly lower hypertension prevalence. The median age of those testing positive was lower than in the negative. The MRI data, acquired before the pandemic, indicated that median liver fat (PDFF) and BMI were higher in people who later tested positive during the COVID-19 pandemic. Although these trends were not significant, the percentage of positive patients who required hospitalization due to COVID-19 presented with significantly higher BMI and liver fat than those positive patients not requiring hospitalization (Figure 2).

**Table 1:** Participant characteristics – data reported as median [interquartile range] or *n* (%). Significance assessments of the negative test group vs. the positive test group, and the positive hospitalized group vs. the positive non-hospitalized group.

	**All positives**	**All negatives**	**Positives & hospitalized**	**Positives & non-hospitalized**
Sample size	1,043	3,415	32	1,011
Median age (IQR)	62 (12.5)	69 (12)^***^	68.5 (17.25)	62 (12)
Gender N (% male)	511 (48.99%)	1,650 (48.31%)	25 (78.12%)	486 (48.07%)^**^
Self-reported white British *N* (%)	921 (88.30%)	3,097 (90.68%)^*^	27 (84.37%)	894 (88.42%)
Diabetes (diagnosed by a doctor) *N* (%)	26 (2.49%)	120 (3.51%)	1 (3.12%)	25 (2.47%)
Hypertension (diagnosed by a doctor) *N* (%)	164 (15.72%)	813 (23.80%)^***^	7 (21.87%)	157 (15.52%)
Median BMI (IQR)	26.26 (5.32)	26.45 (5.18)	28.91 (7.60)	26.21 (5.21)^*^
Liver fat, measured as PDFF (%)	3.27 (4.07)	3.15 (3.33)	5.5 (9.09)	3.24 (3.98)^**^
Liver fibroinflammation, measured as cT1 (ms)	698 (73.25)	698 (72.62)	698 (72)	698 (74)
Previous alcoholic LD *N* (%)	1 (0.09%)	5 (0.14%)	0 (0%)	1 (0.09%)
Previous other LD *N* (%)	15 (1.43%)	84 (2.45%)	1 (3.12%)	14 (1.38%)

Significance is indicated by

* p ≤ 0.05,

** p ≤ 0.01,

*** p ≤ 0.001.

*BMI, body mass index; IQR, interquartile range; LD, liver disease*.

Thirty-two (3.1%) patients in the “positive” group were hospitalized either 1 week or 1 month after the positive test result. These patients were mostly male and had significantly higher BMI and liver fat compared to other positive patients who did not require hospitalization (Table 1). The patients admitted to ICU (*n* = 8) presented with significantly higher liver fat, liver inflammation and BMI compared to all other positive participants who did not require ICU (Table 2). A similar trend was found for those who died after infection (*n* = 14, Table 3).

**Table 2:** Distribution of liver biomarker metrics and BMI in UKB participants who were admitted to ICU 1 week before or 1 month after the COVID-19 test result.

	**ICU participants with a positive test result**	**Non-ICU participants with a positive test result**	**sig**
Sample size	8	1,035	-
Median BMI (IQR)	29.61 (2.99)	26.24 (5.33)	0.037^*^
Median PDFF (IQR)	11.73 (10.32)	3.26 (3.98)	0.003^**^
Median cT1 (IQR)	743 (87.25)	698 (74)	0.0201^*^

Significance is indicated by

* p ≤ 0.05,

** *p ≤ 0.01. BMI, body mass index; PDFF, proton density fat fraction; IQR, interquartile range*.

**Table 3:** Distribution of liver biomarker metrics and BMI in UKB participants who died.

	**Deceased participants with a positive test result**	**Non deceased participants with a positive test result**	**sig**
Sample size	14	1,029	-
Median BMI (IQR)	28.35 (3.29)	26.21 (5.37)	0.009**
Median PDFF (IQR)	4.96 (3.94)	3.26 (4.08)	0.1386
Median cT1 (IQR)	732.5 (49.5)	698 (74)	0.0874

Significance is indicated by ***p ≤ 0.001. BMI, body mass index; PDFF, proton density fat fraction; IQR, interquartile range.

### Univariate and Multivariate Analysis of the Imaging Biomarkers and Known Exposures Linked to COVID-19 Infection

The positive participants had higher liver fat (*p* = 0.0021), were younger (*p* < 0.001), were of non-Caucasian ethnicity (*p* = 0.026) and had lower socio-economic status (Townsend Index) (*p* = 0.015) all of which significantly increased the odds of testing positive for COVID-19. Male gender (*p* = 0.024), young age (< 0.0001), no diagnosis of hypertension (*p* = 0.005), and liver fat ≥10% (0.0025) were significant after stepwise multivariate analysis and define the participants with a positive test result in our population.

### Univariate and Multivariate Analysis of the Imaging Biomarkers and Known Exposures Linked to COVID-19 Hospitalization

The univariate results revealed that being male (*p* = 0.001), of older age (*p* = 0.0007), with high BMI (*p* = 0.004), liver fat higher than 10% (*p* = 0.003) and having lower socio-economic status (Townsend Index) (*p* = 0.04) all significantly increased the odds of being hospitalized with a positive test result for COVID-19. Male gender (*p* = 0.008), older age (*p* = 0.004), high BMI (*p* = 0.021) and having lower socio-economic status (*p* = 0.025) where all significant in the stepwise multivariate analysis with odds ratios of 3.05 (CI: 1.34–6.92), 1.08 (CI: 1.03–1.13), 1.08 (CI: 1–1.7), 1.13 (CI:1.02–1.26), respectively.

### Relationship of BMI and Liver Fat in COVID-19 and Their Association With Being Hospitalized With a Positive Result

**Figure 3:**
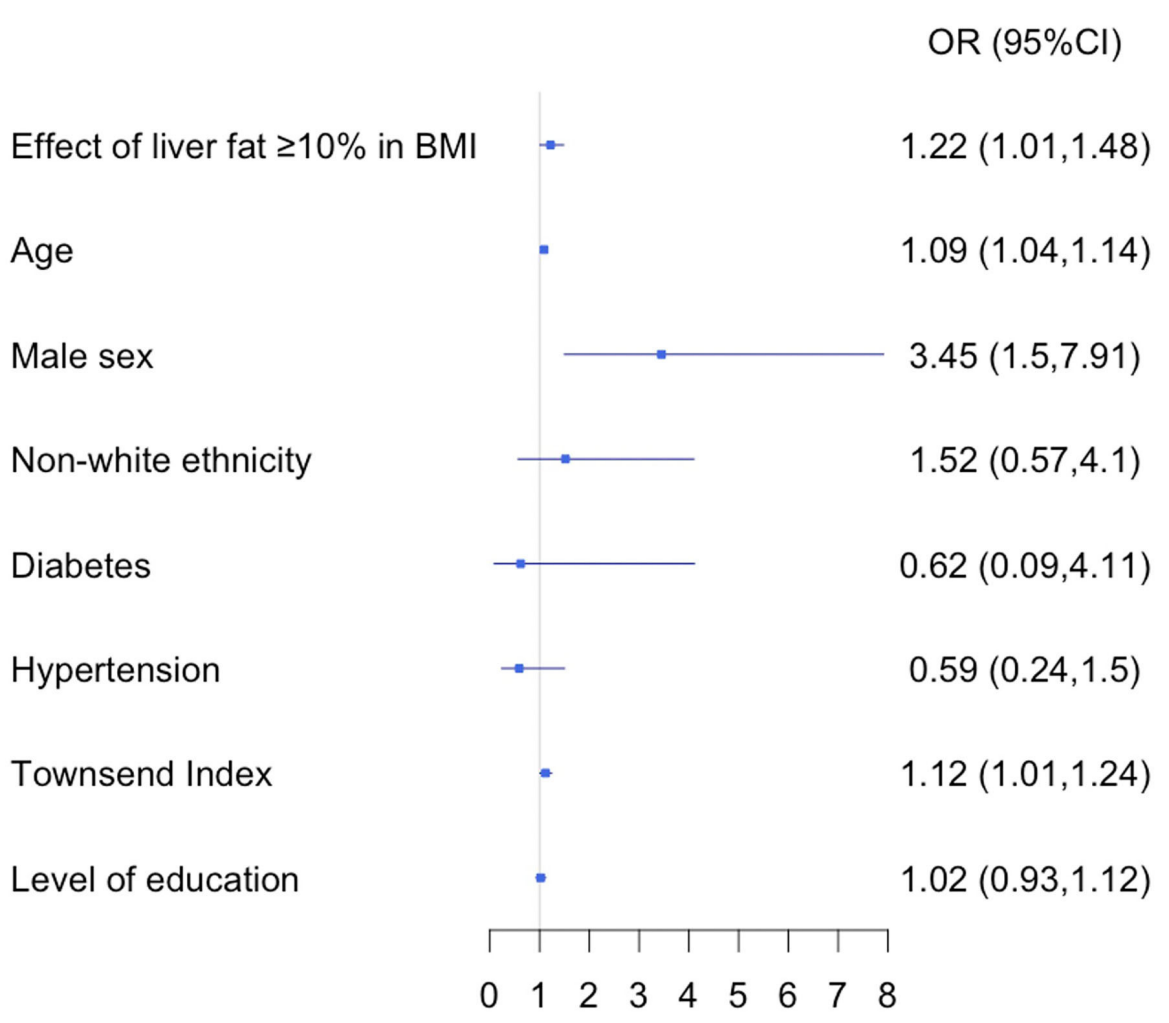
Multivariate stepwise logistic regression, discriminators of being positive hospitalized participants vs. positive non-hospitalized. OR, odds ratio; CI, confidence interval; BMI, body mass index.

**Figure 4:**
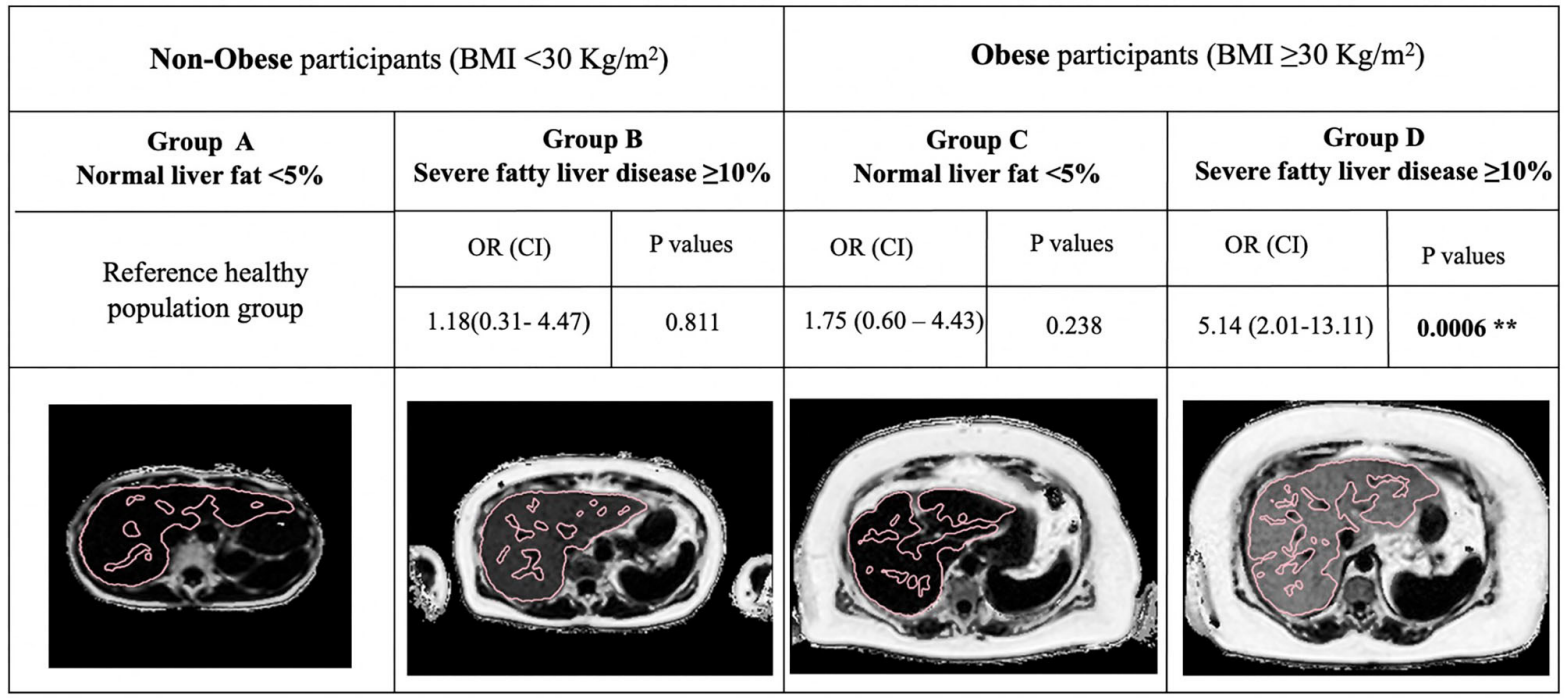
Representative LiverMultiScan images from the UKB cohort, showing multivariate analysis of the “hepato-metabolic risk” variable. BMI <30 Kg/m^2^ and Liver fat <10% was used as reference healthy population. Odds ratio and confidence intervals of the associations between obesity and liver fat are presented for hospitalized with a positive test result participants vs. non-hospitalized positive patients. BMI, body mass index; OR, odds ratio; CI, confidence interval. Significance is indicated by **p ≤ 0.01. Reproduced by kind permission of UK Biobank^©^.

This interaction increased the odds of being hospitalized 1.22 times (CI: 1.01–1.48, *p* = 0.041) (Figure 3), however, the same analysis did not show significance for BMI and low liver fat (≤ 10% PDFF) (odds: 1.25 (CI: 0.99–1.57), *p* = 0.08). The “hepato-metabolic risk” variable in a multivariate setting (Figure 4) revealed that in addition to being male of older age and from a low socio-economic status, participants in group D were at 5.31 times more risk [CI: 2.01–13.11, *p* = 0.0006] of being hospitalized than those on the reference group A. This was not significant in any of the other risk groups.

## Discussion

The aim of this study was to explore the hypothesis that pre-existing liver disease increases the risk of experiencing severe COVID-19 disease. We report on a cohort of 41,791 participants from the UKB with complete liver imaging, of which 4,458 have COVID-19 test results available with 1,043 participants testing positive. Our study has confirmed some of the previously reported risk factors for contracting the disease as being a male or coming from deprived areas. In addition, this work shows that having pre-existing severe fatty liver disease is a significant predictor for testing positive for COVID-19 (*p* = 0.002).

Looking at the risk of being hospitalized with acute COVID-19; a definite profile of older males from deprived socio-economic status that are at higher risk of hospitalization with acute disease emerges, where the presence of metabolic fatty liver disease, as indicated by liver fat ≥10% with BMI ≥ 30 kg.m^2^, is also a significant risk factor for hospitalization with acute COVID-19. To put that into context, 4,655 people in this cohort of 41,791 had severe fatty liver disease, suggesting that 11% of the UK population carries this higher risk for COVID-19.

Several risk factors have been identified from early studies in China (1), Europe (2) and the US (3) that lead to severe infection, including increased age, male sex, non-white ethnicity, and the presence of pre-existing co-morbidities, such as cardiovascular and metabolic conditions, including diseases of the liver. Unlike previous studies, our research did not find increasing age as a risk factor in this cohort, but this is likely an artifact of the limited age range in the UKB population and increasing likelihood of exposure to the virus in people of working age vs. those that had recently retired.

Previous studies have reported BMI as a risk factor for COVID-19, but the mechanism(s) of excess weight in conferring additional risk remained unclear. In this research, those patients with metabolic fatty liver disease defined by liver fat ≥10% and BMI ≥ 30 Kg/m^2^ were 5.14 times more at risk of being hospitalized with severe disease (21). In the UKB imaged population, 7.76% of the obese participants had normal liver fat and were not at increased risk of being hospitalized. This highlights the role of liver fat, in addition to obesity, in the severity of COVID-19. Due to the low sample size available for ICU admissions or death rates, advanced predictive models were not possible. However, patients admitted in the intensive care unit, showed significantly higher BMI, liver fat, and liver fibro-inflammation compared to those hospitalized in the ward supporting the previous statement regarding the important role of liver disease in the context of COVID-19 infection. Moreover, participants who died after a COVID-19 positive test result showed significantly higher BMI compared to those who did not, further research with bigger sample sizes will be key to explore exposures related to the risk of death in more detail.

A major implication of our findings is that it provides impetus and rationale for additional studies to explore the relationship between liver disease and COVID-19, particularly since elevated transaminase levels have also been reported in those presenting with COVID-19 (22). Given the route of cellular entry for SARS-CoV-2 (23) *via* ACE2 receptors, one hypothesis for the mechanistic link between underlying liver disease and increased risk of contracting the disease may be upregulation of the angiotensin pathway and ACE2 receptors in the epithelial receptors in liver.

A potential causal relationship between hepatic steatosis and risk of severe COVID-19 disease should highlight, and motivate, the importance of those at higher risk avoid contracting COVID-19 *via* social distancing and vaccination, but to also the importance of “de-fatting” the liver to reduce susceptibility. Fortunately, recent studies have demonstrated the benefits of aggressive lifestyle interventions (24), and potential new therapeutics that can reverse fatty liver disease in a matter of weeks (25, 26). This may be an important public health message regarding lifestyle choices to reduce your risk as we learn to live with the virus and adapt the new variants over the coming years. Data is also emerging that suggests up to 10% of those who contract COVID-19 may have longer term persistent symptoms (ref), being referred to as Long COVID. Whilst data from this sub-population are not yet available from the UKB database, pre-existing liver disease may also be an important predictor of those more susceptible to long COVID, which would certainly warrant further investigation.

The main limitation of our study is the relatively small proportion of UKB participants for which testing results are available, and the restriction in terms of data available, mainly for blood biomarkers of liver disease. Subsequent analyses as more data are released will provide additional power to investigate a larger number of exposures.

This research provides further evidence on the link between fatty liver disease and the risk of infection and hospitalization with COVID-19. In obese participants, an increased risk of symptomatic disease was not observed in patients without concomitant fatty liver. This highlights the value of identifying this high-risk subpopulation with non-invasive imaging to guide clinical and lifestyle interventions and has the potential to inform public health policy and research priorities around the management of this at-risk population.

## Data Availability Statement

The data analyzed in this study is subject to the following licenses/restrictions: Data belongs to UK Biobank. Requests to access these datasets should be directed to access@ukbiobank.ac.uk.

## Ethics Statement

The studies involving human participants were reviewed and approved by 11/NW/0382. The patients/participants provided their written informed consent to participate in this study.

## Author Contributions

AR-F: data analysis, data curation, and writing the first draft of the manuscript. AD and RN: data analysis and critical input to final manuscript. JM: data curation and critical input to final manuscript. MK: data interpretation and critical input to final manuscript. RB: funding, data interpretation, and critical input to final manuscript. AB: clinical input to data interpretation and critical input to final manuscript. AS: clinical input to data analysis, data interpretation, critical input to final manuscript. All authors contributed to the article and approved the submitted version.

## Conflict of Interest

Perspectum Ltd is a privately funded commercial enterprise that develops medical devices to address unmet clinical needs, including LiverMultiScan^®^. RB is the CEO and founder of Perspectum. AR-F, AD, RN, JM, and MK are employees of Perspectum. AS: None for this project. AS is President of Sanyal Biotechnology and has stock options in Genfit, Akarna, Tiziana, Indalo, Durect Inversago and Galmed. He has served as a consultant to Astra Zeneca, Nitto Denko, Conatus, Nimbus, Salix, Tobira, Takeda, Jannsen, Gilead, Terns, Birdrock, Merck, Valeant, Boehringer-Ingelheim, Bristol Myers Squibb, Lilly, Hemoshear, Zafgen, Novartis, Novo Nordisk, Pfizer, Exhalenz and Genfit. He has been an unpaid consultant to Intercept, Echosens, Immuron, Galectin, Fractyl, Syntlogic, Affimune, Chemomab, Zydus, Nordic Bioscience, Albireo, Prosciento, Surrozen. His institution has received grant support from Gilead, Salix, Tobira, Bristol Myers, Shire, Intercept, Merck, Astra Zeneca, Malinckrodt, Cumberland and Novartis. He receives royalties from Elsevier and UptoDate.

## Abbreviations

ACE2: angiotensin-converting enzyme 2
ALT: alanine transaminase
AST: aspartate aminotransferase
BMI: body mass index
CI: confidence interval
COVID-19: coronavirus disease
cT1: corrected T1
GGT: gamma-glutamyl transferase
GLM: general linear model
MAFLD: metabolic fatty liver disease
MR: magnetic resonance
MREC: North West Multi-Centre Research Ethics Committee
MRI: magnetic resonance imaging
NAFLD: non-alcoholic fatty liver disease
NASH: non-alcoholic steatohepatitis
OR: odds ratio
PDFF: proton density fat fraction
SARS-CoV-2: Severe acute respiratory syndrome coronavirus 2
SD: standard deviation
UKB: UK Biobank.
